# Robotic retroperitoneal lymph node dissection for testicular cancer at a national referral centre

**DOI:** 10.1002/bco2.149

**Published:** 2022-03-31

**Authors:** Anna Grenabo Bergdahl, Marianne Månsson, Göran Holmberg, Magnus Fovaeus

**Affiliations:** ^1^ Department of Urology, Institute of Clinical Science Sahlgrenska Academy at the University of Göteborg Göteborg Sweden; ^2^ Department of Urology Region Västra Götaland, Sahlgrenska University Hospital Gothenburg Sweden

**Keywords:** germ cell tumour, lymph node surgery, retroperitoneal lymph node dissection, robotic retroperitoneal lymph node dissection, RPLND, testicular cancer

## Abstract

**Objectives:**

We aim to determine if robot‐assisted retroperitoneal lymph node dissection (R‐RPLND) can be performed as a safe option to open RPLND in selected patients with metastatic germ cell cancer.

**Patients and methods:**

This population‐based prospective study was performed at a one of two national referral centres for RPLND in Sweden. All patients referred during January 2017–March 2021 were screened for possible inclusion. R‐RPLND was performed using the Da Vinci Xi surgical system. Perioperative parameters, postoperative complications (Clavien–Dindo), final pathology, preservation of antegrade ejaculation and relapse rates were evaluated. Classifiers for selecting patients to open versus robotic RPLND were analysed by logistic regression modelling. The median follow‐up was 23 months.

**Results:**

Of 87 patients referred, 29 were selected for R‐RPLND, 19 in a post‐chemotherapy setting. In median, retroperitoneal tumour diameter was 18 mm, BMI 24 kg/m^2^, operative time 433 min, estimated blood loss 50 ml and length of stay 3 days. One patient underwent open conversion due to failure to progress. Four patients had Clavien–Dindo grade 3 complications, of which three were chylous‐related. No in‐field recurrences occurred during follow‐up.

**Conclusion:**

This population‐based study suggests that R‐RPLND can be safely performed in at least one third of patients referred for an RPLND. A relatively high rate of lymph‐leakage may represent a potential drawback. Tumour size may be the most important discriminator when deciding on robotic versus open RPLND. Further studies with longer follow‐up are needed to validate the results.

## 1 INTRODUCTION

As a part of the National Cancer Strategy in Sweden, several processes have been on‐going during the last decade to improve the quality of cancer care and to provide more equal access to advanced treatments.^1^ In 2013, the Swedish Government and the Swedish Association of Local Authorities and Regions agreed on a plan to promote a national concentration of 10 highly specialized cancer treatments. One of the 10 identified treatments was RPLND in GCT patients. Following an application procedure, two centres were appointed a national commission in 2017: The Department of Urology at Sahlgrenska University Hospital (SUH) in Gothenburg and the Department of Urology at Karolinska University Hospital, Huddinge in Stockholm. A national multidisciplinary team (MDT) conference was established to aid in adherence to clinical guidelines and to improve the cooperation between professions at a national level.

The role of RPLND in patients with metastatic nonseminomatous germ cell cancer (NSGCT) and residual masses after chemotherapy is well established.^2^, ^3^ RPLND is a technically challenging procedure traditionally performed through open surgery. In advanced cases with large tumours surrounding the aorta and vena cava, vascular reconstructions and synchronous organ resections may be warranted. There is however a proportion of patients with very limited metastatic growth that require an RPLND. In addition, primary RPLND in low‐volume metastatic seminoumatous GCT (SGCT) is currently being evaluated in two trials.^4^, ^5^

A concentration of RPLND cases to SUH brought opportunities for research and technology development. At the time, only a limited number of small R‐RPLND case series had been published. However, the short‐term results were promising, and R‐RPLND was considered feasible in selected patients.^6^, ^7^, ^8^ SUH has a long experience of robot‐assisted surgery including ~400 urologic robotic procedures yearly. After ethical approval, we started a prospective R‐RPLND study in 2017. The rationale was to prospectively explore if R‐RPLND could be implemented safely without compromising the oncologic efficacy. The primary aim was to evaluate perioperative and postoperative outcome of R‐RPLND including complications and relapse rates. The secondary aim was to provide guidance on how to select patients to R‐RPLND.

## 2 PATIENTS AND METHODS

### 2.1 Overview

All Swedish GCT patients considered for an RPLND are discussed in a weekly national MDT conference where urologists, oncologists, radiologists and pathologists are represented. Initial (pre‐chemotherapy) imaging and post‐chemotherapy imaging are presented. When needed, testicular or nodal biopsy histology are demonstrated by the attending pathologist. After decision to proceed with an RPLND, patients are referred to one of the two NRCs. In the present study, we screened all patients referred to SUH from 1 January 2017 until 1 March 2021 for possible inclusion in the R‐RPLND study. Patients with limited nodal disease (<50 mm diameter) and no suspicion of tumour‐infiltration of major vessels were considered for robotic surgery. Potential risks and benefits were discussed with the patient, and written informed consent was obtained. To be able to analyse selection mechanisms, comparisons with the O‐RPLND patients referred during the same time period were performed. The study was approved by the regional ethics committee (Dnr 418‐17 2017‐06‐29).

### 2.2 Setting

According to the SWENOTECA treatment‐protocol, a post‐chemotherapy RPLND (PC‐RPLND) is indicated in NSGCT patients with a residual mass measuring ≥10 mm in largest transverse diameter after completion of chemotherapy. Patients with late marker‐negative relapses (>2 years after initial successful treatment for metastatic disease) are also candidates for RPLND. A unilateral template restricted to the primary landing zone of the affected testicle is an option for tumours measuring 10–49 mm, whereas tumours ≥50 mm should prompt bilateral templates. In addition, location of enlarged nodes must be considered before deciding template to make sure that all areas with enlarged nodes on pre‐chemotherapy and post‐chemotherapy radiology are excised. A primary (without induction‐chemotherapy) RPLND (P‐RPLND) is recommended in patients with: (a) persistent marker‐negative NSGCT clinical stage (CS) IIA disease; (b) pure teratoma in the testicle and limited metastatic disease; and (c) CS I post‐pubertal teratoma with malignant somatic de‐differentiation. A P‐RPLND has recently also become a recommended option in SGCT CS IIA‐B with 1–2 retroperitoneal nodes, 10–30 mm in largest diameter.^9^ A nerve‐sparing unilateral resection is recommended in P‐RPLND. A detailed description of templates has been published by the SWENOTECA group previously.^10^

All R‐RPLNDs were performed by two surgeons (M. F. and A. G. B.), both with significant experience in open and robotic retroperitoneal surgery. The da Vinci Xi System (Sunnyvale, CA, USA) was used, with a lateral flank approach in the initial unilateral cases. Later, we changed to a 20‐degree supine Trendelenburg position, with four 8‐mm robot‐ports placed in a linear configuration infra‐umbilically and one 12‐mm assistant port as described by others.^7^, ^11^ No re‐docking was needed in any of the cases.

### 2.3 Primary outcomes

Patient demographics (age, BMI and ASA), tumour characteristics (testicular pathology and largest transverse diameter of retroperitoneal nodes), clinical stage, IGCCCG risk‐group classification,^12^ and previous chemotherapy regimens were recorded in a study‐database. Surgical field (unilateral/bilateral), operative time (OT), estimated blood loss (EBL), length of stay (LOS) and final pathology was recorded. Following discharge, all patients received a follow‐up phone call by the operating surgeon within 3 months. Complications and occurrence of retrograde ejaculation were asked for and registered. Medical charts were also checked for complications in all patients within 90 days after surgery. Surgical complications were graded according to the Clavien–Dindo classification system, (complication grade 3a or higher considered ‘major’). All patients received continuous oncological follow‐up according to the SWENOTECA cancer care programme^9^ and were monitored using the national quality register (NQR) for testicular cancer.^13^

### 2.4 Secondary outcomes

No explicit predefined criteria were used to select patients to robotic/open RPLND other that the general principle that high volume residual disease probably is more suitable for open surgery whereas small residuals without vascular infiltration can be suited for robotics. The plan was to explore the selection mechanism in retrospect to provide guidance for future use.

### 2.5 Analysis

Descriptive data was presented as frequencies and median (inter quartile range, IQR). Follow‐up time was calculated from R‐RPLND date until the last clinical follow‐up date according to the medical charts and NQR. OT, EBL and LOS were compared between different template R‐RPLNDs, and between O‐RPLND and R‐RPLND using the Mann–Whitney *U*‐test. The exploration of selection mechanisms was done by means of logistic regression models of the type of surgery actually carried out, using retroperitoneal tumour diameter, risk‐group, induction‐chemotherapy (yes/no), tumour histology, ASA, BMI (<30 or ≥30), and age as explanatory variables. Area under the ROC (receiver operating characteristics) curves (AUC) was calculated, models compared by means of likelihood ratio tests, and cut‐offs explored. No adjustment for multiple comparisons was performed.

## 3 RESULTS

Of 87 patients admitted for an RPLND at SUH during the study period, 29(33%) were selected for robotic surgery. R‐RPLND patients had a more favourable disease and smaller retroperitoneal tumours both pre‐chemotherapy and pre‐RPLND than O‐RPLND patients (good risk in 93% vs. 59%; median residual tumour size 18 mm vs. 28 mm). There were more SGCT patients among the R‐RPLND cases, whereas almost all O‐RPLND patients had NSGCT or advanced extragonadal tumours. All patients had abdominal nodal involvement with at least one node ≥10 mm at time of surgery (CS ≥IIA) (Table 1).

**TABLE 1:** Patient demographics and clinical characteristics of all RPLND patients admitted to Sahlgrenska University Hospital from 1 Jan 2017 until 1 March 2021

Patient and tumour characteristics	Robot‐assisted RPLND, *N* = 29			Open RPLND, *N* = 58		
	Median (IQR)	Mean (range)	*N* (%)	Median (IQR)	Mean (range)	*N* (%)
Age, years	33 (29–37)	35 (18–62)		33 (26–47)	37 (17–74)	
BMI	24 (22–28)	24 (16–35)		24 (22–28)	26 (18–42)	
National referral^c^			18 (62)			29 (50)
Regional referral^d^			11 (38)			29 (50)
Surgical indication						
*Post‐chemotherapy*			18 (62)			48 (83)
*Late relapse* ^g^			6 (21)			8 (14)
*Primary RPLND CS* ≥ *IIA*			3 (10)			2 (3.4)
*Lumpectomy, seminoma*			2 (6.9)			0
Chemotherapy before RPLND						
*Yes*			19 (66)			50 (86)
*No*			10 (34)			8 (14)
Testicular tumour histology						
*Non Seminoma*			20 (69)			45 (78)
*Seminoma*			6 (21)			1 (1.7)
*Teratoma*			1 (3.4)			3 (5.2)
*Malignant transformation*			1 (3.4)			0
*Benign (extragonadal)*			1 (3.4)^a^			6 (10)^b^
*Burned out*			0			3 (5.2)
Retroperitoneal tumour size, mm						
*Pre‐chemotherapy*	23 (17–30)	29 (11–104)	18 (62)	46 (33–72)	55 (8–206)	50 (86)
*Pre‐RPLND*	18 (15–26)	22 (11–50)	29 (100)	28 (20–53)	48 (8–217)	58 (100)
Abdominal stage^e^						
*A (1* to *<2 cm)*			17 (59)			14 (24)
*B (2 to <5 cm)*			11 (38)			29 (50)
*C (5 to<10 cm)*			1 (3.4)			8 (14)
*D (>10 cm)*			0			7 (12)^12^
Prognostic group^f^						
*Good*			26 (93)			34 (59)
*Intermediate*			1 (3.6)			11 (19)
*Poor*			1 (3.6)			13 (22)
*Missing*			1			0

Abbreviations: BMI, body mass index; IQR, interquartile range; RPLND, retropertioneal lymph node dissection.

^a^ Extragonadal seminoma.

^b^ Extragonadal tumours with yolk sac tumour (*n* = 2), teratoma (*n* = 1), malignant transformation of teratoma (*n* = 1) and necrosis/fibrosis (*n* = 2) in the resected retroperitoneal nodes.

^c^ Referrals from outside the regional catchment‐area.

^d^ Intra‐regional referrals.

^e^ Abdominal stage at time of staging following diagnosis or recurrence.

^f^ International Germ Cell Cancer Collaborative Group risk‐group at time of staging.

^g^ >2 years after initial management (chemotherapy or surveillance) and complete remission.

Of the 29 robot‐cases, two with SGCT were scheduled for a lumpectomy only and therefore excluded from the separate analysis of the R‐RPLND cases. The total R‐RPLND study population comprised 27 patients undergoing a unilateral (*n* = 23, 85%), or a full bilateral (*n* = 4, 15%) template resection. Clinical details are presented in Table 2. Regarding primary tumour pathology, 19 patients had NSGCT, four had SGCT, two had teratoma and one had malignant transformation to adenocarcinoma. One patient had a primary extragonadal biopsy‐verified SGCT and no testicular tumour.

**TABLE 2:** Perioperative data of robot‐assisted and open RPLND procedures

Perioperative parameters and complications	Unilateral R‐RPLND, *N* = 23		Bilateral R‐RPLND, *N* = 4		All R‐RPLND, *N* = 27		O‐RPLND, *N* = 58
	Median (IQR)	Count	Median (IQR)	Count	Median (IQR)	Count	Median (IQR)
Tumour size pre‐RPLND, mm	17 (14–25)		23 (18–27)		18 (15–27)		26 (20–53)
Operative time, min	420 (335–469)		494 (449–653)		433 (375–470)		297 (230–440)
Estimates blood loss, ml	50 (25–150)		100 (75–200)		50 (25–150)		400 (300–1000)
Length of stay, days	3.0 (2.0–6.0)		3.5 (3.0–6.0)		3.0 (2.0–4.0)		7.0 (5.0–9.0)
Antegrade ejaculation							
*Yes*		10		2		12	N/A
*No*		2		2		4	N/A
*Missing*		11		0		11	N/A
Lymph node yield, no	13 (7.0–15)		27 (15–43)		13 (8.0–19)		15 (8.0–23)
Open conversion		1		0		1	N/A
Postoperative complications							
CD3	3		1		4		9
CD4	0		0		0		2
CD5	0		0		0		2

Abbreviations: CD, Clavien–Dindo classification system; N/A, not applicable; O‐RPLND, open retroperitoneal lymph node dissection; R‐RPLND, robot‐assisted retroperitoneal lymph node dissection.

The most common surgical indication among the R‐RPLND patients was a residual mass after completion of chemotherapy for NSGCT (*n* = 18, 67%). Of the remaining patients, five had a late relapse after median (IQR) 6.3 years (5.6–6.9) following initial chemotherapy (*n* = 3, 11%) or initial surveillance (*n* = 2, 7.4%). Four CS IIA patients underwent a P‐RPLND; two due to marker‐negative SGCT, one due to teratoma only in the testicle and a growing paraaortic cystic mass, and one due to malignant transformation of teratoma in the testicle and an enlarged aortocaval node.

### 3.1 Perioperative outcomes and complications in R‐RPLND

The median OT varied with extent of resection, left‐sided resections took significantly less time than both right‐sided and bilateral resections (median 380 min vs. 435 and 495 min, respectively; *p* = 0.02 and *p* = 0.01), but no significant difference was observed between right‐sided and bilateral resections. The corresponding mean OT (range) was 390 min (158–592) for unilateral resections, 551 min (435–780) for bilateral resections, and 551 min (435–780) for all resections. Median LOS was 3 days, with no significant difference between left/right/bilateral resections. Median EBL was 50 ml for left‐ and right‐sided resections and 100 ml for bilateral resections, but the difference was not statistically significant. Compared with O‐RPLND, the median LOS and EBL was significantly improved in R‐RPLND patients (3 vs. 7 days; *p* < 0.01, 50 vs. 400 ml; *p* < 0.01). One patient underwent open conversion due to failure to progress (an obese patient with BMI 35 kg/m^2^). No adjunctive procedures (i.e., organ resections/vascular reconstructions) were performed in any R‐RPLND patient although one patient had an inferior mesenteric artery injury that was repaired robotically.

There were four major postoperative complications in four R‐RPLND patients. One had a herniation of mesenteric fat at a port‐site requiring surgical reposition under general anaesthesia. Three (11%) of the R‐RPLND patients had chylous‐related complications (compared with four [6.9%] among the O‐RPLND cases). All three had undergone a PC‐RPLND. One recovered after percutaneous drainage only, but two were put on TPN and subsequently reoperated after 2 and 3 months, respectively, with closure of leaking lymph vessels. Both recovered quickly afterwards. There were no Clavien–Dindo grade 4 or 5 complications among the R‐RPLND cases (Table 2).

### 3.2 Pathological, oncological and functional outcome in R‐RPLND

Pathology of the resected specimen are presented in Table 3. Positive nodes were detected in 17 cases (63%); 12 had teratoma (44%) and 5 viable cancer (19%). In post‐chemotherapy resections, the rate of teratoma/fibrosis was 50%/50%; none had viable GCT. All relapse patients had positive nodes at R‐RPLND, three with viable GCT (of which two received adjuvant chemotherapy) and two with teratoma. During a median (IQR) follow‐up time of 23 (8.6–29) months, no in‐field relapses occurred among the R‐RPLND patients, but there were two out‐of‐field recurrences; one poor prognosis patient relapsed retrocrurally (surgically managed openly at our institution), and one patient with malignant transformation of teratoma had a supraclavicular nodal relapse (treated with chemotherapy). In the open group, five (8.6%) out‐of‐field recurrences was observed. Regarding ejaculatory function, data were available in 16 cases only. Of those, 12 (75%) had preserved antegrade ejaculation. The four patients reporting retrograde ejaculation were all post‐chemotherapy NSGCT patients; two had undergone bilateral resections and one had undergone open conversion. When excluding the patient who in fact underwent an O‐RPLND, the rate of antegrade ejaculation was 80%.

**TABLE 3:** Pathological outcome following R‐RPLND

Pathological outcome	Type of R‐RPLND			Total
	Primary	PC	Late relapse	
Seminoma	1		3	4
Malignant transformation of teratoma	1			1
Teratoma	1	9	2	12
Benign/necrosis	1	9		10
*Pathological N stage*				
pN0	1	9		10
pN1		4	3	7
pN2	3	5	1	9
pN3		0	1	1

Abbreviations: PC, post‐chemotherapy; pN stage, pathological nodal stage; R‐RPLND, robot assisted retroperitoneal lymph node dissection.

### 3.3 Selection of patients

According to the univariable logistic regression models, retroperitoneal tumour diameter was the single most valuable classifier to discriminate between R‐RPLND and O‐RPLND cases (AUC 0.71), followed by prognostic group (AUC 0.67), testicular histology (AUC 0.62) and chemotherapy (AUC 0.60). Due to the correlation between prognostic group and tumour diameter, prognostic group did not add any statistically significant value to a model with tumour diameter alone. However, when testicular histology was added to tumour diameter in the model, the AUC increased to 0.77 (*p* = 0.02). BMI, age and ASA were poor classifiers (AUC 0.55, 0.52 and 0.54, respectively, in univariable models) (Table S1).

**FIGURE 1:**
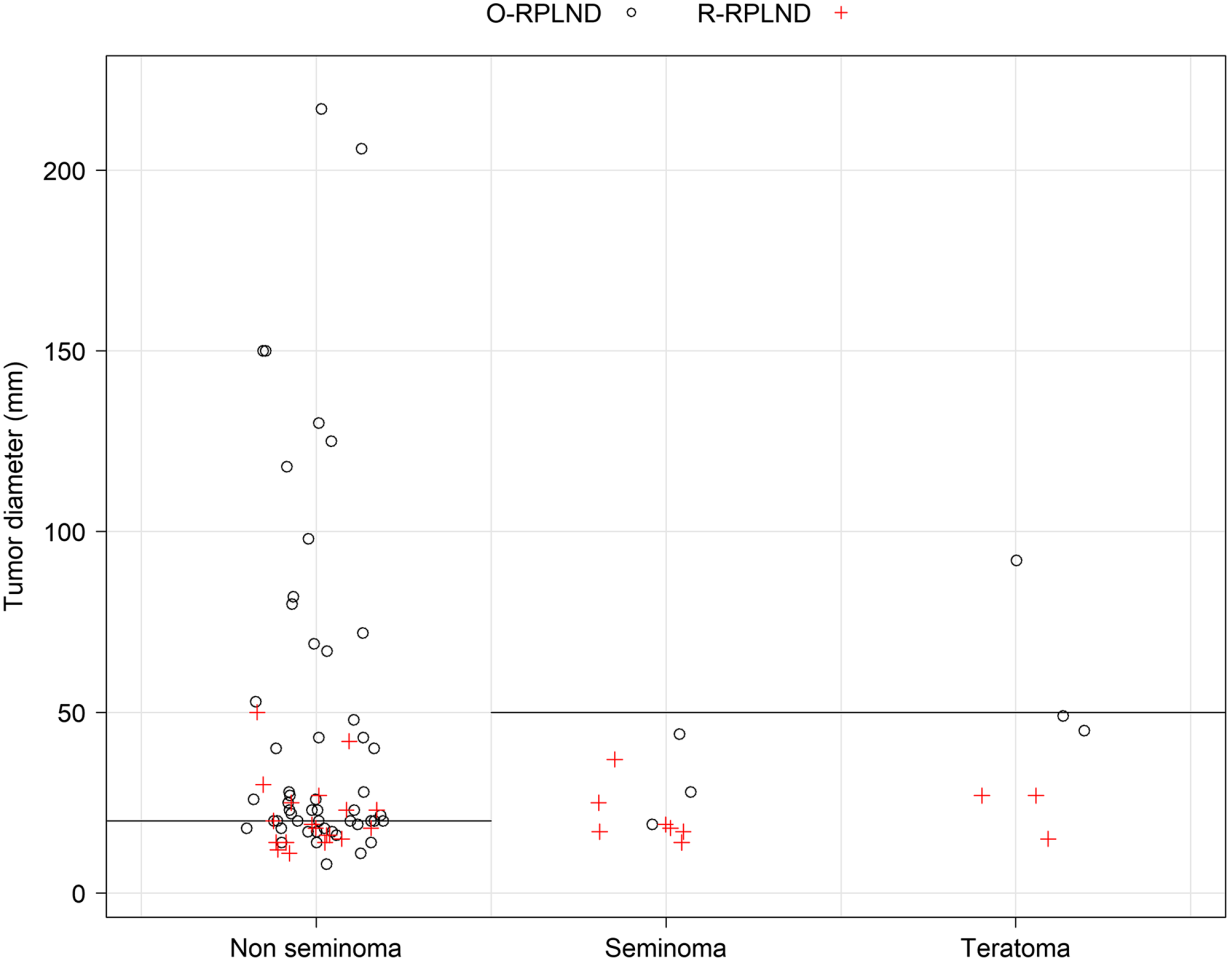
Plot of testicular histology and retroperitoneal tumour diameter in patients selected for open versus robot assisted retroperitoneal lymph node dissection

The model based on testicular histology and tumour diameter is in essence the same as having different tumour diameter cut‐offs for NSGCT and SGCT/teratoma, respectively. For instance, choosing patients with NSGCT and retroperitoneal tumours <20 mm, or SGCT/teratoma and tumours <50 mm, for robotic surgery, would pick 21 out of 29 robotics, but also include 18 out of 58 open procedures in our data. If the same cut‐off of tumour diameter was used irrespective of histology, the number of incorrectly selected open surgeries raised to 28 while keeping 20 robotics (Figures 1 and S1).

## 4 DISCUSSION

Given the excellent cure rates with today's germ cell tumour (GCT) management, research efforts have increasingly focused on how to decrease treatment‐related sequelae. Open retroperitoneal lymph node dissection (O‐RPLND) is associated with significant morbidity and not all patients benefit therapeutically from it. We report perioperative outcome and mid‐term oncological results from a NRC for RPLND after 5 years' experience of R‐RPLND. So far, 29 patients with CS > I GCT have undergone an R‐RPLND, with only one open conversion and no in‐field recurrences after median 23 months of follow‐up. With the advantage of a significantly shorter LOS and reduced EBL, R‐RPLND seems an option in good prognosis cases with CS 2A/B disease but should be restricted to high volume centres with expertise in open and robotic RPLND.

The first R‐RPLND case was reported in 2006 by Davol on a patient with NSGCT in the testicle and a negative CT of the chest and abdomen.^14^ Most series published thereafter have predominately included CS I patients undergoing primary R‐RPLND, and the study design has been retrospective. To our knowledge, this is the first prospective study on CS > I R‐RPLND patients. The majority were in the post‐chemotherapy setting; only nine were P‐RPLND. However, three of those nine were treated with chemotherapy at time of diagnosis >2 years earlier. Consequently, only six patients were chemotherapy‐naïve in this study.

It is well‐known that primary resections have a lower complication rate than post‐chemotherapy resections. The latter are more complex due to the chemotherapy‐induced desmoplastic reaction. In this study of mainly small volume disease in the post‐chemotherapy setting, we observed four major complications. Three were in post‐chemotherapy patients and chylous‐related, of which two were surgically managed. This incidence of chylous ascites was higher than anticipated, although small in number (11% vs. 6.9% in robot‐ and open RPLND, respectively). Previous studies have pointed towards a tendency of increased incidence of chylous ascites with minimally invasive RPLND, and induction chemotherapy may increase the risk.^15^, ^16^ Singh et al. reported a chyle leak rate of 31% in 13 post‐chemotherapy R‐RPLND and hypothesized that a lesser use of suturing and clipping of lymphatics compared with O‐RPLND played a role.^17^ In our study, we used Hem‐o‐Lok clip ligation meticulously. Yet, when the two chyle‐leak cases were explored, a diffuse leak from a tangle of clips near the renal hilum was observed. Whether the clips prevented the tissue from adhering, or whether major lymphatics had been left unsealed remains unknown. Percutaneous lymphatic embolization has become increasingly used to treat postoperative lymph‐leakage.^18^, ^19^, ^20^ To date, those facilities are not readily available at our institution.

Apart from chylous ascites, another concern that has been raised regarding R‐RPLND has been unusual patterns of disease recurrence.^21^ We noted two out‐of‐field recurrences, although not in unusual places. Hence, we have no reason to believe that this was inherent to the robotic technique but longer follow‐up is needed to monitor areas of potential recurrences.

The main advantages with minimally invasive RPLND are perhaps the short LOS and the low EBL, and possibly also the preservation of antegrade ejaculation. In a recent review of R‐RPLND including eight series with >10 patients in each, the reported weighted means regarding EBL was 132 ml, and LOS 2 days.^14^ This is in comparison with our results (median EBL 118 ml and LOS 3 days), given the differences in patient characteristics among open and robotic cases. Previous reports on preserved ejaculatory function vary, from 85% to 100% of cases,^6^, ^16^, ^17^, ^22^, ^23^ to somewhat lower rates, 67%–81%.^24^, ^25^ We were unable to assess ejaculatory function in 11 patients, but 80% of the remaining R‐RPLND patients reported antegrade function.

While striving towards decreasing overtreatment and reducing therapy‐related side effects, it is important to recall that we lack diagnostic tools to tell whether a residual mass contains cancer, teratoma or fibrosis. We know from large O‐RPLND studies of post‐chemotherapy NSGCT patients that the rate of teratoma in resected specimen is ~40%, and viable cancer is 11%–17%.^26^, ^27^, ^28^, ^29^, ^30^ The remaining large proportion of patients undergo the procedure without an immediate clinical benefit although the risk of relapse decreases.^31^ In our study of mixed post‐chemotherapy and primary cases, 63% had teratoma or viable cancer in the resected specimen. This relatively high rate indicates a fair selection of patients despite smaller tumours compared with the open series.

How to best select RPLND patients to robotics is an unsettled issue. The short follow‐up in this study warrants caution in interpreting the results. However, the low complication rate and excellent oncological results (no in‐field recurrences despite a 63% rate of positive nodes and a low rate of adjuvant chemotherapy; *n* = 2, 7.4%) points towards an appropriate patient selection. As expected, the statistical modelling used to analyse the selection mechanism suggests that tumour diameter was the most important factor in our selection. Furthermore, the modelling suggests that tumour histology mattered. Note that the model should be regarded as an objective description of how the selection looked in the present study rather than as a recommendation, which would need a larger study and a model validation. When reviewing those that were incorrectly classified as robot cases in the example, the vast majority were unsuitable for robotics due to tumour localization (suprahilar/retrocrural); hence location is another factor that we believe needs to be considered. Contrary to what was anticipated, the BMI was a poor predictor of being selected to O‐RPLND versus R‐RPLND (AUC 0.55). However, the one conversion was in an obese patient.

It is important to recall that we selected small‐volume infrahilar disease to R‐RPLND, as opposed to clearly unsuitable cases with large‐volume disease growing diffusely close to great vessels, renal hilum, bowel, and vertebrae. To further evaluate the selection of patients to either approach, and to possibly suggest a future selection model for clinical use, R‐RPLND should be restricted to high volume centres with expertise in open RPLND and robotic surgery so that more data can be prospectively collected.

The strength of this study was the prospective design and the reasonably large patient volumes that comes with a national commission. The drawback of concentrating patients to NRCs is that it might be difficult to follow patients over time. It is possible that complications were caught to a higher degree in patients residing in the Gothenburg area. However, all patients are presented again at the national MDT conference when the pathology report is available, and this MDT serves as an additional follow‐up of post‐discharge complications. The NQR's high coverage also adds to our belief that all significant surgical complications and recurrences were detected. Another weakness was the lack of validated assessment tools for patient reported outcome measures including evaluation of ejaculatory function, especially with respect to the distinction between a nerve‐sparing procedure and a unilateral template resection.

## 5 CONCLUSION

According to these population‐based results, at least one third of all RPLNDs in GCT patients may be performed as a robotic procedure at a high‐volume centre without jeopardizing oncological safety. R‐RPLND has the potential to decrease the burden of treatment‐related side‐effects although a higher than anticipated chyle‐leak may be a concern. How to best select patients to open versus robot‐assisted RPLND needs further evaluation and analysis with longer follow‐up, but our results indicate that retroperitoneal tumour size may be the single most important determinant.

## AUTHOR CONTRIBUTIONS

All authors made significant contributions to this work. AGB wrote the manuscript and secured funding. MF, GH and MM contributed to the design, the statistical analyses and helped to draft the manuscript. AGB (robot and open), MF (robot) and GH (open) performed all surgical procedures.

## Supporting information


**Figure S1.** Supporting Information

**Table S1.** Supporting Information
