# Supplementary figures and images for: Robotic retroperitoneal lymph node dissection for testicular cancer at a national referral centre
Source: BJUI Compass. 2022 Mar 31;3(5):363–70. doi: 10.1002/bco2.149 (PMC9349583; doi:10.1002/bco2.149)

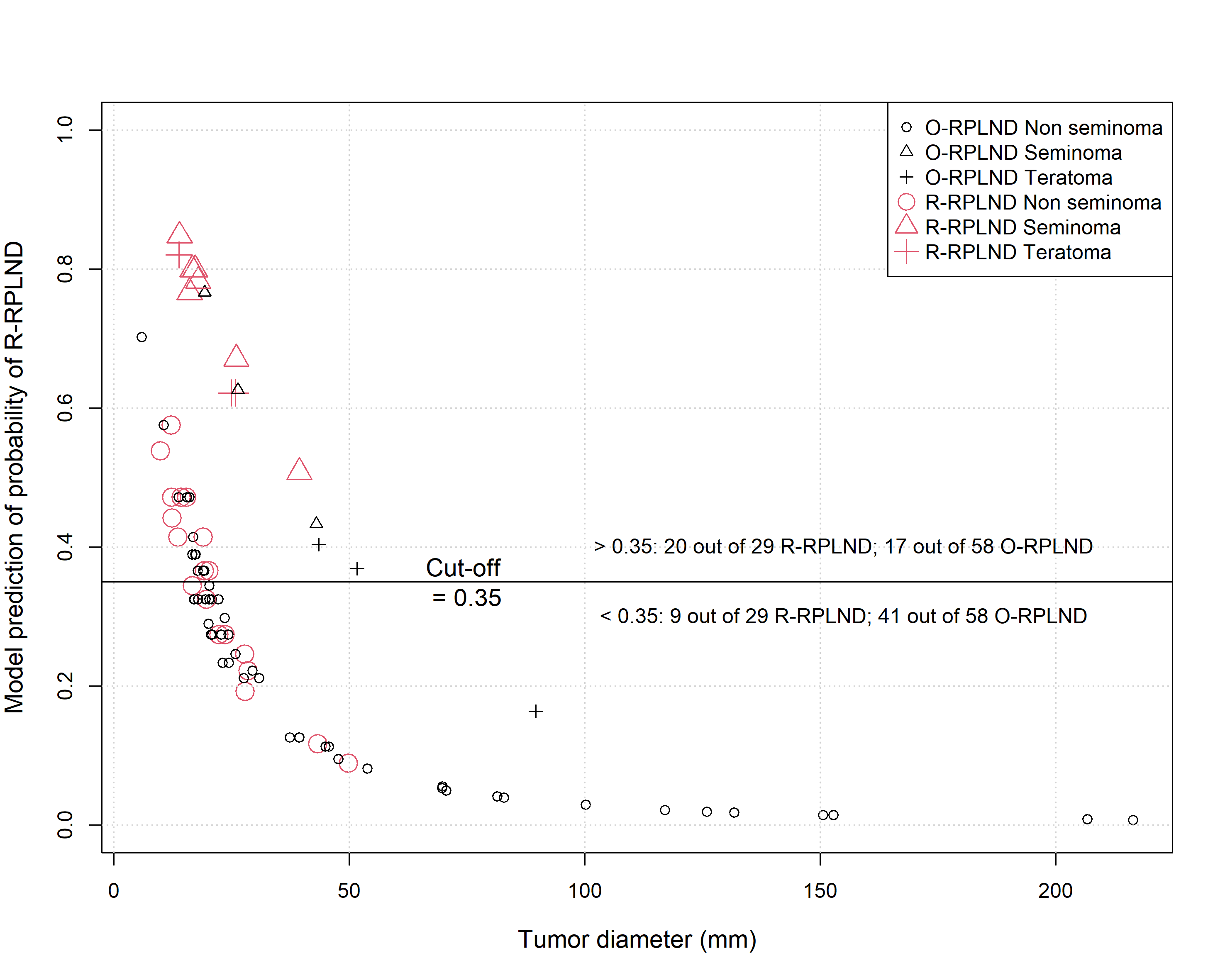

Supplement: Supplementary file 1 — Figure S1. Supporting Information [file BCO2-3-363-s002.tiff]
