# Supplementary material for: Robotic retroperitoneal lymph node dissection for testicular cancer at a national referral centre
Source: BJUI Compass. 2022 Mar 31;3(5):363–70. doi: 10.1002/bco2.149 (PMC9349583; doi:10.1002/bco2.149)
Supplement: Supplementary file 2 — Table S1. Supporting Information [file BCO2-3-363-s001.docx]

**Table 4. (***Supplemenatary)*

|  | *Univariable analysis* | | *Multivariable analysis*  *Tumor diameter + Testicular histology* | |
| --- | --- | --- | --- | --- |
|  | AUC (95% CI) | OR, robot vs open (95% CI) | AUC (95% CI) | OR, robot vs open (95% CI) |
| *Tumor*  *diameter* | 0.71 (0.60-0.82) | Doubling of diameter: 0.36 (0.16-0.66) | 0.77 (0.67-0.88)  (p = 0.02 vs tumor diameter alone, LR test) | Doubling of diameter: 0.30 (0.12-0.62) |
| *Testicular*  *histology* | 0.62 (0.53-0.72) | SGCT vs NSGCT: 6.4 (1.6-32)  Teratoma vs NSGCT: 2.7 (0.47-16) |  | SGCT vs NSGCT: 6.2 (1.5-34)  Teratoma vs NSGCT: 5.8 (0.8-48) |
| *Prognosis*  *group* | 0.67 (0.59-0.75) | Cat 2 vs 1: 1.2 (0.04-32)  Cat 3 vs 1: 9.9 (1.8-186) |  |  |
| *Chemotherapy* | 0.60 (0.50-0.70) | No vs yes: 3.3 (1.1 – 9.9) |  |  |
| *BMI* | 0.55 (0.48-0.62) | ≥30 vs <30: 0.36 (0.05-1.5) |  |  |

AUC=Area under the curve

OR=Odds ratio

CI=Confidence interval

SGCT=Seminomatous germ cell tumor

NSGCT=Nonseminomatous germ cell tumor

BMI=Body mass index

Cat 1=Good prognosis group

Cat 2=Intermediate prognosis group

Cat 3=Poor prognosis group
